# Differences in stress coping between individuals from Germany and American Samoa and their relation to obesity, perceived stress, and depressive symptoms

**DOI:** 10.1186/s41043-025-01099-4

**Published:** 2025-11-06

**Authors:** Anne Schrimpf, Micah van der Ryn, Stephen T. McGarvey, Arno Villringer, Michael Gaebler

**Affiliations:** 1 Institute for General Practice, Faculty of Medicine, Leipzig University, Philipp-Rosenthal-Straße 55, 04103 Leipzig, Germany; 2 Community and Natural Resources, American Samoa Community College, Mapusaga, American Samoa; 3 Department of Epidemiology and International Health Institute, School of Public Health, Brown University, Providence, RI USA; 4 Department of Anthropology, Brown University, Providence, RI USA; 5 Max Planck Institute for Human Cognitive and Brain Sciences, Leipzig, Germany; 6 Leipzig University Medical Center, IFB Adiposity Diseases, Leipzig, Germany; 7 Clinic of Cognitive Neurology, University Hospital Leipzig, Leipzig, Germany; 8 Berlin School of Mind and Brain, MindBrainBody Institute, Humboldt- University, Berlin, Germany; 9 Leipzig Research Center for Civilization Diseases (LIFE), Leipzig University, Leipzig, Germany

**Keywords:** Cross-cultural comparison, Pacific islands, Stress, psychological, Eating disorders, Depression, Mental health, Coping skills

## Abstract

Stress and how individuals cope with it can have substantial implications for health. As the socio-cultural environment influences individual perceptions of stress and coping behavior, differences between cultural groups may have different effects on physical and mental health. Using qualitative interviews and questionnaires, this study examined cultural differences in stress coping and their interplay with perceived stress (Perceived Stress Questionnaire, Trier Inventory for Chronic Stress), BMI (normal-weight, obesity), and depressive symptoms (Beck Depression Inventory Short Form) in young adults from Germany (individualistic society, *n* = 120) and American Samoa (collectivistic society, *n* = 56). Stress coping strategies were assessed through interviews, after which the data were quantified as problem-focused, emotion-focused, or avoidance coping. Group differences in coping strategies were analyzed using Gamma generalized linear models, group differences in perceived stress and depressive symptoms were analyzed using univariate ANCOVAs. The results showed that the German group used more problem-focused coping, while the Samoan group used more avoidance coping. The use of avoidance coping was associated with higher perceived stress in the German group but with lower perceived stress in the Samoan group. In general, depressive symptoms were higher among participants from the Samoan than the German group. However, Samoan individuals with obesity who used avoidance coping had lower depressive symptoms than Samoan individuals with obesity who did not. This difference was not observed in the German group. Irrespective of cultural background, individuals with obesity tended to use more avoidance and less emotion-focused coping, and to report eating more during stress compared to individuals with normal-weight. On the one hand, our findings may contribute to a better understanding of how the cultural environment influences stress coping and its potential adaptive effects on perceived stress and depressive symptoms, highlighting the need for culturally appropriate stress management interventions. On the other hand, the association between weight status and avoidance coping might be relatively stable across cultures, offering opportunities for intervention.

## Introduction

The impact of socio-cultural factors on health outcomes has been widely recognized in public health. In particular, stress and how individuals cope with it can have a significant impact on their health. While the link between stress coping strategies and obesity (e.g., Varela et al., [1, 2] as well as depressive symptoms [3] has been widely studied, the role of cultural differences in stress coping strategies and health risks remains an area of ongoing research. As the socio-cultural environment influences both the individual perception of stress as well as coping behavior [4, 5], these differences between cultural groups can have varying effects on physical and mental health [6, 7]. For instance, certain coping strategies have been found to correlate either differentially or to varying degrees with depressive symptoms in different cultural groups [8, 9]. Further, although the relationship between depressive symptoms and obesity is well described [10], there is evidence that this effect might not be present in all ethnic groups [11]. It is therefore conceivable that the perception of stress in individuals with obesity and its impact on mental well-being might differ between cultural groups. Overall, cultural differences in stress coping and their potential interactions with obesity, perceived stress, and depressive symptoms are multifaceted and have rarely been investigated.

Stress has been described as interaction between an individual and a stressful situation, in which there is a discrepancy between the perceived demands and the ability to adjust [12]. This implies that the (negative) impact of stress can be moderated by the individual’s coping resources such as the cognitive and behavioral efforts to manage stress [12]. Lazarus described two, often intertwined ways of managing stress: *problem-focused* and *emotion-focused* coping [12]. The former is directed at altering the source of stress (e.g., generating and enacting solutions, confrontation), while the latter is directed at regulating the emotional response to stress (e.g., seeking emotional support and self-control). In addition to changing the situation (problem-focused) or the self (emotion-focused), *avoidance* (e.g., ignoring or withdrawing from the source of stress) has been identified as another independent coping response [13, 14]. Individual coping strategies boost or buffer the impact of a stressful event: for instance, emotion-focused coping might increase anxiety during stress, whereas problem-focused coping could improve the outcome of the stressful event [15]. Avoidance coping, on the other hand, might promote maladaptive habits, such as addictions or overeating [16, 17]. However, these studies were mainly conducted in Western societies and might not reflect a general pattern.

Socio-cultural background, values, and norms provide direction for behavior [4] and influence which coping patterns are legitimate, appropriate, and valued in a society [12, 14]. Specifically, on a relative spectrum, members of more collectivistic (higher social conformity and obligation towards the social and physical environment) and more individualistic (higher individual autonomy and control over the social and physical environment) societies might experience different pressures or demands: individuals from more individualistic societies might face more responsibilities to take care of themselves, whereas those from more collectivistic societies might have stronger obligations to their family or community [4]. Accordingly, individuals in more collectivistic compared to more individualistic environments might be less likely to apply problem-focused coping strategies, such as controlling or altering the situation, as this may affect others and hamper the in-group harmony [4, 18, 19]. Instead, they might more frequently employ coping strategies that focus on changing and regulating the self, such as emotion-focused or avoidance coping [18, 20], which has been demonstrated in some cross-cultural studies [8, 14, 21, 22]. Further, cultural differences in stress coping and in perceived stress were shown to be highly adaptive: avoidance coping was associated with psychological symptoms in an individualistic but not in a collectivistic population [6]. Further, perceived support and emotional expressivity buffered the physiological response to evaluative stress in individuals from individualistic but not from collectivistic groups [7]. These findings indicate that the cultural environment, on a relative spectrum of being more or less individualistic/collectivistic, is an essential factor when examining the interactions between stress coping, perceived stress, and mental well-being.

Stress is hypothesized to be a considerable risk factor for the development of overweight and obesity [23]. Although some studies found no or only a weak relationship between perceived stress and body-mass-index (BMI) [24, 25], others found a positive association [23, 26]. These inconsistent findings might be explained by differential cortisol responses mediated by coping strategies: lower stress-related cortisol responses were linked to a stronger sense of mastery, defined as the belief that one’s actions significantly affect the environment [27]. Conversely, an increase in cortisol levels during examination stress was related to both weight gain and a decrease in mastery [28]. One possible mechanism could be that some coping strategies do not effectively regulate the stress response, as they focus on avoidance rather than addressing the stressor. As a result, they may be ineffective at reducing physiological arousal and are more likely to be associated with heightened cortisol secretion. Importantly, elevated cortisol secretion during stress has been associated with increased consumption of high-calorie foods [29] and greater accumulation of abdominal fat [30], linking ineffective stress coping to weight gain. Behavioral research supports these findings: individuals with more avoidant coping during stress have a higher risk of weight gain and uncontrolled eating or eating disorders [1, 2, 16, 17], whereas individuals with adaptive stress management capabilities tend to have lower body weight [31]. In addition to the potential interplay between coping strategies and weight gain, obesity has been modestly related to depressive symptoms [10]. In turn, individuals with depression use more avoidance coping [32], which suggests a potential complex interplay between stress coping, obesity, and depressive symptoms. As cultural differences have rarely been addressed, the relationship between weight gain and avoidant coping might vary across cultural groups. For example, avoidance coping mediated the relationship between perceived stress and eating disorders among White but not Black women in South Africa [33]. Therefore, some individuals might respond to stress by gaining weight, but this effect might be partly mediated by cognitive processes such as stress coping and might differ depending on the cultural environment.

Although differences in stress coping have been found between individuals with and without obesity as well as between cultural groups, it is not clear to what extent the socio-cultural environment influences obesity-related differences in stress coping and how this affects perceived stress and depressive symptoms. The present study was conducted in Germany, a Western high-income and more individualistic society, and American Samoa, a Polynesian, middle income and more collectivistic society. We used a qualitative interview method to examine stress coping strategies during stress among young adults. We also explored the relationship between these coping strategies and obesity status, as well as with stress state and depressive symptoms measured by questionnaires. Our hypotheses are:

1. Based on the literature distinguishing individualistic and collectivistic orientations [8]; O’Connor & Shimizu, [22]; Oláh [14], we hypothesize that individuals from the Samoan group will report a higher use of emotion-focused and avoidance coping strategies compared to individuals from the German group. Conversely, we predict that individuals from the German group will report a higher use of problem-focused coping compared to the Samoan group.
2. In line with research suggesting avoidance coping may contribute to weight gain [1, 2, 17], we hypothesize that individuals with obesity will report more avoidance coping strategies compared to individuals with normal weight. We further explore potential interactions with the cultural background.
3. We hypothesize an association between the coping strategy and perceived stress as well as depressive symptoms. Considering the adaptive nature of coping strategies in different cultural groups [6, 7], we specifically hypothesize to find higher perceived stress and depressive symptoms in Germans but not in Samoans who used avoidance coping. Further, we also expect interactions between coping strategy and weight status.
4. For stress-related eating behavior, defined here as the self-reported tendency to eat more, less, or the same amount of food in response to stress, we hypothesize that individuals with obesity in both cultural samples will be significantly more likely to report an increase in food intake during stress compared to individuals with normal-weight.

## Methods

### Design and procedure

This study employed a cross-cultural, mixed-methods design. Data were collected in two study locations, Germany and American Samoa, to examine location- and obesity-related differences in perceived stress, depressive symptoms, and stress coping strategies. The project was conceived as a comparative study but was executed in two phases: The first phase of data collection was conducted in Germany (from 12/2012 to 03/2014). Following the acquisition of fieldwork funding, the second phase was carried out in American Samoa (from 09/2015 to 12/2015). In both locations, the procedure was identical. Data collection involved quantitative questionnaires (perceived stress, depressive symptoms) and qualitative interviews (stress coping strategies). The data from both locations were subsequently combined for a cross-cultural analysis where ‘location’ (Germany vs. American Samoa) served as the primary independent variable for comparison.

The procedure was as follows: After consenting to participate, a trained experimenter (AS) obtained anthropometric measurements (weight, height, waist and hip circumferences) from the participants. Then, BMI and waist-to-hip ratio (WHR) were calculated (BMI = (kg/m²), WHR = waist circumference in cm/hip circumference in cm). Participants were then classified as either belonging to the obesity group or the normal-weight group; individuals in the overweight category were not included in the study.

All eligible participants then completed validated questionnaires in their native language (German or English) to assess perceived stress and depressive symptoms. Translation equivalence was ensured by using established versions of the questionnaires in both languages. Subsequently, participants took part in a short semi-structured interview designed to explore stress coping strategies and eating behavior during stress in depth. Interviews were conducted in person (by AS), following a standardized guide, and recorded using a digital voice recorder for later transcription and analysis.

### Recruitment of participants

In both locations, a short demographic questionnaire was used to assess eligibility for the study (see the inclusion/exclusion criteria below), asking about age, sex, level of education, and history of psychological or psychiatric treatment. To assign participants to the obesity or normal-weight group according to BMI, the experimenter (AS) measured the weight and height of all potential participants before they enrolled in the study.

For the sample in Germany, participants were recruited from the database of the Max Planck Institute for Human Cognitive and Brain Sciences, Leipzig, Germany. A total of 120 individuals, matched for education and age, were included in the study. Inclusion criteria: Participants had to be between 18 and 35 years of age and have a BMI ≥ 30.0 kg/m^2^ for participants with obesity, or between 18.5 kg/m^2^ and 24.9 kg/m^2^ for participants with normal-weight. Exclusion criteria were a self-reported history of neurological and psychiatric treatment.

For the sample in American Samoa, participants were recruited at the American Samoa Community College. Participants were recruited from the campus area based on availability. A total of 60 individuals, matched for education and age, participated in this study. Inclusion criteria were similar to those in Germany (i.e., participants should be between 18 and 35 years of age). Additionally, participants had to be fluent in English. The majority of participants were of Samoan descent, one participant was of Filipino descent. BMI criteria were adjusted to a recommended cut-off for Polynesian populations, as the body composition of Pacific Islanders consists of a higher percentage of muscle mass and thus differs from that of Europeans with an equivalent BMI [34]: BMI ≥ 32.0 kg/m^2^ for participants with obesity and BMI between 18.5 kg/m^2^ and 27.9 kg/m^2^ for participants with normal-weight. Exclusion criteria were the same as for the German sample. Four individuals with overweight but not obesity were subsequently excluded due to a BMI between 28.0 kg/m^2^ and 31.9 kg/m^2^, leaving 56 participants who were either normal-weight or had obesity in the analyses (detailed sample characteristics for both datasets are shown in Table 1). The two samples are described in detail in Schrimpf et al. [35, 36].

**Table 1:** Sample characteristics

	Germany		American Samoa	
	Individuals with normal-weight *n* = 60	Individuals with obesity *n* = 60	Individuals with normal-weight *n* = 26	Individuals with obesity *n* = 30
***Anthropometrics***				
Age	26.85 ± 3.67	26.88 ± 3.53	20.00 ± 2.00	20.43 ± 1.68
Male/Female	27/33	27/33	11/15	16/14
BMI	21.85 ± 1.71	35.61 ± 4.41	25.49 ± 3.05	41.11 ± 6.19
WHR	0.77 ± 0.06	0.89 ± 0.10	0.78 ± 0.04	0.85 ± 0.07
***Coping strategies***				
Problem-focused	29.55 ± 36.25	23.22 ± 32.45	8.97 ± 23.68	15.78 ± 29.03
Emotion-focused	45.99 ± 36.02	33.69 ± 31.57	48.72 ± 41.14	26.78 ± 35.52
Avoidance	24.44 ± 33.27	39.75 ± 34.51	42.31 ± 40.41	57.44 ± 38.18
***Questionnaires***				
BDI-SF	3.13 ± 3.43	2.85 ± 3.29	7.35 ± 6.83	7.53 ± 6.84
PSQ-20 Total Score	1269 ± 310	1255 ± 294	1574 ± 265	1574 ± 238
PSQ-20 Worries	30.44 ± 19.77	29.56 ± 19.15	58.21 ± 23.21	56.89 ± 23.21
PSQ-20 Tension	33.22 ± 18.84	31.00 ± 17.26	55.13 ± 19.46	52.44 ± 16.68
PSQ-20 Lack of joy	62.89 ± 19.81	61.33 ± 18.93	39.74 ± 19.00	40.22 ± 19.30
PSQ-20 Demands	40.33 ± 20.70	39.11 ± 20.45	49.49 ± 16.35	54.89 ± 17.65
TICS Work overload	14.47 ± 7.15	12.73 ± 6.25	17.04 ± 6.04	16.50 ± 6.97
TICS Social overload	8.07 ± 4.90	9.32 ± 5.96	13.62 ± 4.88	12.03 ± 5.55
TICS Pressure to perform	16.80 ± 6.37	16.42 ± 5.74	21.77 ± 6.20	18.00 ± 8.12
TICS Work discontent	13.30 ± 6.20	11.93 ± 4.68	15.88 ± 5.36	15.90 ± 6.84
TICS Excessive demands at work	6.70 ± 3.58	6.18 ± 4.19	12.31 ± 4.63	11.60 ± 4.56
TICS Lack of social recognition	5.48 ± 3.49	5.10 ± 3.11	7.42 ± 3.35	7.37 ± 3.93
TICS Social tensions	6.65 ± 4.41	6.02 ± 4.34	11.08 ± 5.04	10.27 ± 5.46
TICS Social isolation	7.90 ± 4.00	7.57 ± 4.90	12.77 ± 4.93	12.47 ± 4.70
TICS Chronic worrying	6.88 ± 3.31	5.85 ± 3.63	8.58 ± 3.69	8.13 ± 4.07

Means and standard deviations for anthropometrics, coping strategies, and questionnaire scores. BDI-SF = Beck’s Depression Inventory short form, BMI = Body-mass-index, PSQ-20 = Perceived Stress Questionnaire, TICS = Trier Inventory for Chronic Stress, WHR = Waist-to-hip ratio

### Questionnaires

In both locations, participants completed questionnaires to assess perceived stress and depressive symptoms. The validated English and German versions of the following questionnaires (references are provided in brackets for each version) were employed:

The **Beck Depression Inventory Short Form** (BDI-SF, Beck et al. [37, 38] consists of 13 items and measures the severity of the affective and cognitive components of depression over the past two weeks. Items are rated on a four-point Likert scale and measure the severity of certain feelings experienced by the participant in the past two weeks (0 = no symptoms to 3 = most severe symptoms). The total score ranges from 0 to 39, with a score above 9 identified as the threshold for depressive symptoms. The BDI-SF displayed acceptable to good internal consistency in the current study (Germany: α = 0.79; Samoa: α = 0.89).

The **Perceived Stress Questionnaire** (PSQ-20, Fliege et al. [39, 40] consists of 20 items and measures perceptions of and emotional responses to stress over the past month. A total score as well as four subscales of five items each are assessed: “worries”, “tension”, “lack of joy”, and “demands”. The first three factors represent internal stress reactions, whereas the fourth factor, “demands”, focuses on perceived external stressors. Items are rated on a four-point Likert scale and measure how often a particular experience applies to the participant over the past month (1 = almost never, 2 = sometimes, 3 = often, 4 = usually). Higher values indicate greater stress. The internal consistency of the current samples was only acceptable or good for the PSQ-20 Total Score and PSQ-20 Worries. Therefore, we only included these two scales in the analysis (PSQ-20 Total Score Germany: α = 0.90, Samoa: α = 0.77; PSQ-20 Worries Germany: α = 0.81, Samoa: α = 0.78; PSQ-20 Tension Germany: α = 0.76, Samoa: α = 0.55; PSQ-20 Lack of joy Germany: α = 0.77, Samoa: α = 0.63; PSQ-20 Demands Germany: α = 0.81, Samoa: α = 0.45).

The **Trier Inventory for Chronic Stress** (TICS, Petrowski et al. [41, 42] consists of 57 items and measures perceived chronic stress over the past three months. Nine factors of chronic stress are assessed: work overload, social overload, pressure to perform, work discontent, excessive demands at work, lack of social recognition, social tensions, social isolation, and chronic worrying. Items are rated on a five-point Likert scale and measure how often a particular situation or experience has happened to the participant in the last three months (0 = never, 1 = rarely, 2 = sometimes, 3 = often, 4 = very often). Higher values indicate greater stress. The TICS showed acceptable to good internal consistency in our sample (TICS Work overload Germany: α = 0.90, Samoa: α = 0.83; TICS Social overload Germany: α = 0.85, Samoa: α = 0.79; TICS Pressure to perform Germany: α = 0.82, Samoa: α = 0.86; TICS Work discontent Germany: α = 0.81, Samoa: α = 0.79; TICS Excessive demands at work Germany: α = 0.86, Samoa: α = 0.76; TICS Lack of social recognition Germany: α = 0.82, Samoa: α = 0.71; TICS Social tensions Germany: α = 0.86, Samoa: α = 0.78; TICS Social isolation Germany: α = 0.84, Samoa: α = 0.75; TICS Chronic worrying Germany: α = 0.84, Samoa: α = 0.80). The results for each questionnaire are shown in Table 1.

### Interviews

In both locations, semi-structured interviews were used to assess stress coping strategies, allowing for a culturally sensitive and context-rich evaluation, particularly in a cross-cultural setting. The interview consisted of two main questions: First, the participants were asked about their usual stress coping strategies in acute stressful situations, such as high work load, examinations, or deadlines (1. main question: “First, can you tell me about how you usually cope in stressful situations, such as during high workload or examinations?“). If needed and depending on the participants’ initial answers, open-ended follow-up questions (e.g., “Can you tell me more about that?” or “Can you give me an example?”) were used to elicit more detail. Second, they were asked whether they change their eating behavior during stress (2. main question: “Do you tend to eat more, less, or the same amount of food when you are stressed, e.g., with a high work load?”). The interview lasted approximately 10 min. We subsequently quantified the qualitative data to enable statistical comparison between groups, identify patterns and prevalence, and explore the relationship between the coping styles identified in the interviews and the depression/perceived stress scores from the questionnaires.

### Coding of the interviews

To compare coping strategies between Germans and Samoans, and between individuals with and without obesity, a numerical coding approach was selected to align with the study’s comparative aims and to allow integration of interview-derived coping data with quantitative questionnaire measures. Participants’ recorded statements were transcribed verbatim, the transcripts were re-read several times, and the content was analyzed using deductive content analysis [43]: To examine recurring themes and identify categories in the interviews, statements were categorized either as *problem-focused* coping, *emotion-focused* coping, or *avoidance* coping. The definitions of these coping strategies were derived from previous research [13, 44, 45] and are shown in Table 2. As many participants scored in more than one coping category, we calculated the percentage of all statements that were assigned to each coping dimension (number of statements per category/number of total statements*100). Examples of participants’ quotes for each of the three categories are shown in Table 2. The coding was carried out by two researchers, who were both blind to the participants’ BMI status or sex. The location could not be blinded due to the different languages used during the interviews. Reliability of the coding was ensured by an inter-coder agreement (> 90%) based on a second researcher who repeated 60% (randomly chosen) of the coding independently.

**Table 2:** Stress coping coding scheme and examples of participants’ stress coping strategies assigned to the three categories

Coping category	Definition	Example quotes
Problem-focused	Changing the source of stress Confronting the source of stress Planning (thinking about next steps or strategies) Increasing effort Seeking instrumental support (such as advice or help) Suppress competing activities	*“I just organize everything on a piece of paper or in my calendar and then I just cross out everything one by one. I try to manage my time wisely and then I just do the work.”* (Samoan man with obesity, 20) *“I prepare myself*,* face the situation*,* and get through it.”* (German woman, normal-weight, 29)
Emotion-focused	Talking about the source of stress (emotional support) Changing the self Controlling emotions Acceptance (learning to live with it) Positive reframing Humor (jokes, making fun) Venting (expressing negative emotions) Religion (praying, meditating) Restraint Activities to enhance mood	*“When I am home I just listen to music or go to sleep. That’s what I do when my sisters are not around*,* but I usually talk to my sisters.”* (Samoan woman, normal-weight, 20) *“I try to calm myself down and encourage myself that this time will pass at some point. I also speak with friends.“* (German man, normal-weight, 27)
Avoidance	Ignoring, avoiding, or leaving the source of stress Denial Substance use (alcohol, drugs, food) Behavioral and mental disengagement Self-distraction (getting involved in other activities)	*“Usually*,* when I am really stressed out*,* I tend to eat junk food and when I eat junk food I can’t control myself. I always kind of eat the stress away. It kind of helps to cope with what’s going on.”* (Samoan woman with obesity, 23) *“When I can’t get around the stress*, e.g.,* during exams*,* I try to ignore the stress and refuse to get involved.”* (German man, normal-weight, 26)

### Statistical analysis

All statistical analyses were carried out using IBM SPSS Statistics 27 (Armonk, NY, USA) with a two-sided α-level of 0.05. Continuous variables were presented as mean (M) ± standard deviation (SD). Group differences in coping strategies were analyzed using Gamma generalized linear models (GLM) with log link and with the between-subject factors “weight status” (normal-weight, obesity) and “location” (Germany, American Samoa) and the covariate “age” and “sex” (male, female). This robust approach was chosen due to the non-normal, right-skewed distribution of the coping strategy data and its ability to accommodate categorical predictors.

To analyze potential interaction effects with “weight status” and “location”, the continuous variables problem-focused coping, emotion-focused coping, and avoidance coping (indicating the percentage) were binarized in a next step (i.e., coding whether (> 0%) or not (= 0%) a participant was using this strategy). Then, group differences in perceived stress and depressive symptoms (BDI, PSQ-20, TICS) were analyzed using univariate ANCOVAs with between-subject factors “weight status”, “location”, and “coping” (no, yes), and with the covariate “age” and “sex” (male, female). For all ANCOVAs, estimated effect sizes are reported using partial eta squared (η_p_^2^). If ANCOVAs showed a significant main effect or interaction, least significant differences tests were used to determine the origin and direction of the effect, in which case we report M ± SD. For all ANCOVAs, the Benjamini-Hochberg false discovery rate method (FDR) was used for multiple comparison corrections.

For the categorial variable “eating during stress” (more, less, equal amount), chi-square tests were used to test for associations with “weight status”. Estimated effect sizes were reported using Cramer’s V.

## Results

A comprehensive overview of the descriptive statistics for each measure can be found in Table 1. All scales reported there were analyzed. However, to focus on the most robust findings, the Results section only reports effects that remained statistically significant after applying FDR correction.

### Sample characteristics

Means and standard deviations for sample characteristics of each group can be found in Table 1. The samples were significantly different in age (main effect of location: *F*(1, 174) = 170.629, *p* <.001, η_p_^2^ = 0.495), because the Samoan sample was younger (*M* = 20.23, *SD* = 1.83) than the German sample (*M* = 26.87, *SD* = 3.59). The samples also differed in BMI (main effect of location: *F*(1, 174) = 14.863, *p* <.001, η_p_^2^ = 0.079), showing a higher BMI in the Samoan (*M* = 33.85, *SD* = 9.28) compared to the German sample (*M* = 28.73, *SD* = 7.67). No significant differences in WHR were found between the two locations (*F*(1, 174) = 0.740, *p* =.391, η_p_^2^ = 0.004).

### Coping strategies

**Fig. 1:**
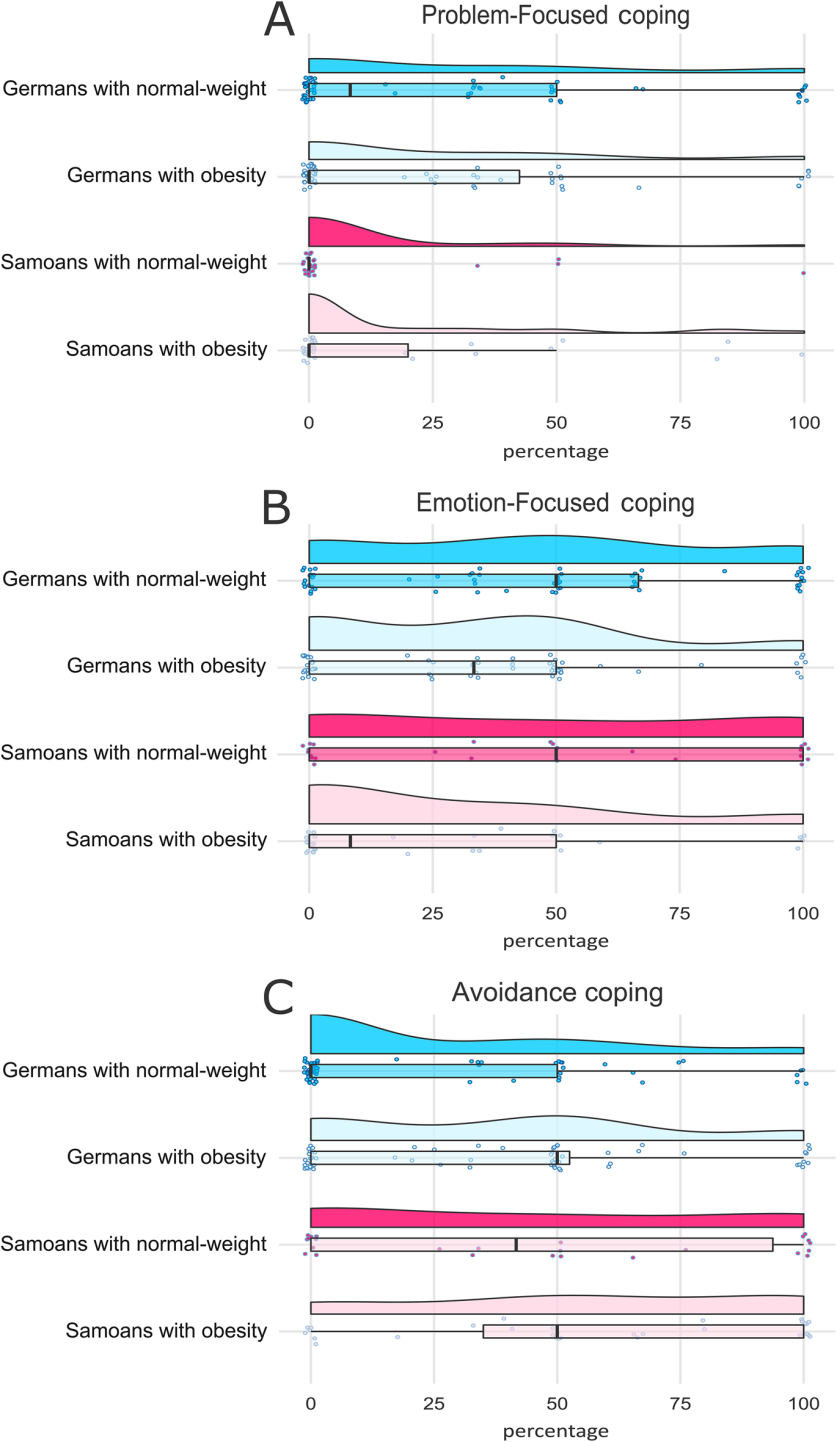
Distribution of stress coping strategies used by German and American Samoan participants, stratified by weight status. The y-axis denotes the four participant subgroups (Germans with normal-weight, Germans with obesity, Samoans with normal-weight, Samoans with obesity). The x-axis represents the percentage of each participant’s total coping statements that were assigned to a specific coping category. Each panel displays a different coping strategy. Within each subgroup, the data distribution is shown using violin plots and boxplots. Each dot represents the score for an individual participant. Panel (**A**): The use of problem-focused coping strategies differed significantly between the two locations, with the German group (*M* = 26.39, *SD* = 34.41) reporting more problem-focused coping than the Samoan group (*M* = 12.62, *SD* = 26.66; Wald χ^2^(1) = 8.679, *p* =.003). Panel (**B**): The use of emotion-focused coping strategies differed significantly between weight status groups, with individuals with normal-weight (*M* = 46.82, *SD* = 37.42) reporting more emotion-focused coping than individuals with obesity (*M* = 31.39, *SD* = 32.90; Wald χ^2^(1) = 8.668, *p* =.003). Panel (**C**): The use of avoidance coping strategies differed significantly between the two locations and the two weight status groups, with individuals with obesity (*M* = 45.65, *SD* = 36.54) reporting more avoidance coping than individuals with normal-weight (*M* = 29.84, *SD* = 36.29; Wald χ^2^(1) = 5.480, *p* =.019), and the Samoan group (*M* = 50.42, *SD* = 39.61) reporting more avoidance coping than the German group (*M* = 32.10, *SD* = 34.62; Wald χ^2^(1) = 7.347, *p* =.007)

The use of *problem-focused* coping strategies differed significantly between the two locations, indicating a greater usage of problem-focused coping in the German group (*M* = 26.39, *SD* = 34.41) compared to the Samoan group (*M* = 12.62, *SD* = 26.66; main effect of location: Wald χ^2^(1) = 8.679, *p* =.003, Fig. 1A). No significant main effect of weight status (Wald χ^2^(1) = 0.325, *p* =.569) or interaction between location and weight status (Wald χ^2^(1) = 2.222, *p* =.136) were found.

The analysis of *emotion-focused* coping strategies revealed a significant main effect of weight status (Wald χ^2^(1) = 8.668, *p* =.003). Independent of location, individuals with normal-weight more frequently used emotion-focused coping (*M* = 46.82, *SD* = 37.42) than individuals with obesity (*M* = 31.39, *SD* = 32.90, Fig. 1B). No significant main effect of location (Wald χ^2^(1) = 0.305, *p* =.581) or interaction between location and weight status (Wald χ^2^(1) = 0.858, *p* =.354) were found.

The analysis of *avoidance* coping strategies showed significant main effects of weight status (Wald χ^2^(1) = 5.480, *p* =.019) and location (Wald χ^2^(1) = 7.347, *p* =.007). Individuals with obesity more frequently used avoidance coping (*M* = 45.65, *SD* = 36.54) than individuals with normal-weight (*M* = 29.84, *SD* = 36.29) and Samoans more often used avoidance coping (*M* = 50.42, *SD* = 39.61) than Germans (*M* = 32.10, *SD* = 34.62, Fig. 1C). No significant interaction between location and weight status (Wald χ^2^(1) = 0.270, *p* =.603) was found.

### Coping strategies and perceived stress measured by TICS and PSQ-20

The use of *problem-focused* and *emotion-focused* stress coping was not significantly associated with perceived stress (all *ps*_FDR_ >0.05).

**Fig. 2:**
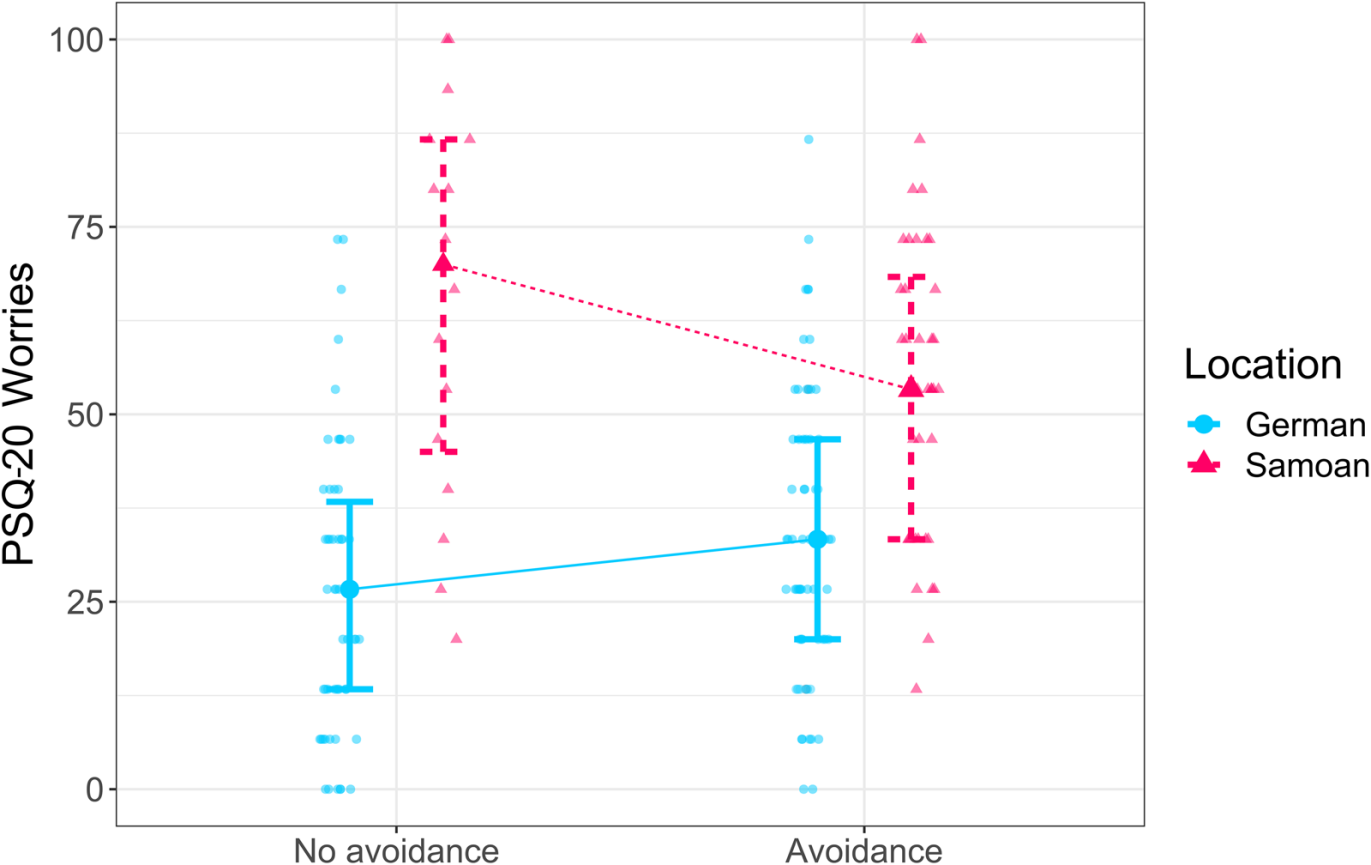
In the German group, the use of avoidance coping was associated with more worrying (*M* = 33.33, *SD* = 19.04) compared to Germans who did not use avoidance coping (*M* = 25.93, *SD* = 19.19). This difference was reversed in the Samoan group, showing that Samoans exhibited more worries when not using avoidance (*M* = 65.42, *SD* = 26.24) compared to Samoans who were using avoidance coping (*M* = 54.33, *SD* = 21.11; *F*(1, 166) = 9.044, *p* =.003, η_p_^2^ = 0.052). PSQ-20 = Perceived Stress Questionnaire

The analyses of *avoidance* stress coping showed that Samoans who were using avoidance coping reported lower perceived lack of social recognition (*M* = 6.83, *SD* = 3.28) compared to Samoans who were not using avoidance coping (*M* = 8.81, *SD* = 4.20). This effect was absent in the German group (interaction of location*avoidance coping with TICS lack of social recognition: *F*(1, 166) = 6.236, *p* =.013, η_p_^2^ = 0.036). Further, worries were lower in Germans who were not using avoidance coping (*M* = 25.93, *SD* = 19.19) compared to Germans with avoidance coping (*M* = 33.33, *SD* = 19.04), whereas Samoans exhibited more worries when not using avoidance (*M* = 65.42, *SD* = 26.24) compared to Samoans who were using avoidance coping (*M* = 54.33, *SD* = 21.11; interaction of location*avoidance coping with PSQ-20 worries: *F*(1, 166) = 9.044, *p* =.003, η_p_^2^ = 0.052, Fig. 2).

### Coping strategies and depressive symptoms measured by BDI-SF

The use of *problem-focused* and *emotion-focused* stress coping was not significantly associated with the severity of depressive symptoms (all *ps*_FDR_ >0.05).

**Fig. 3:**
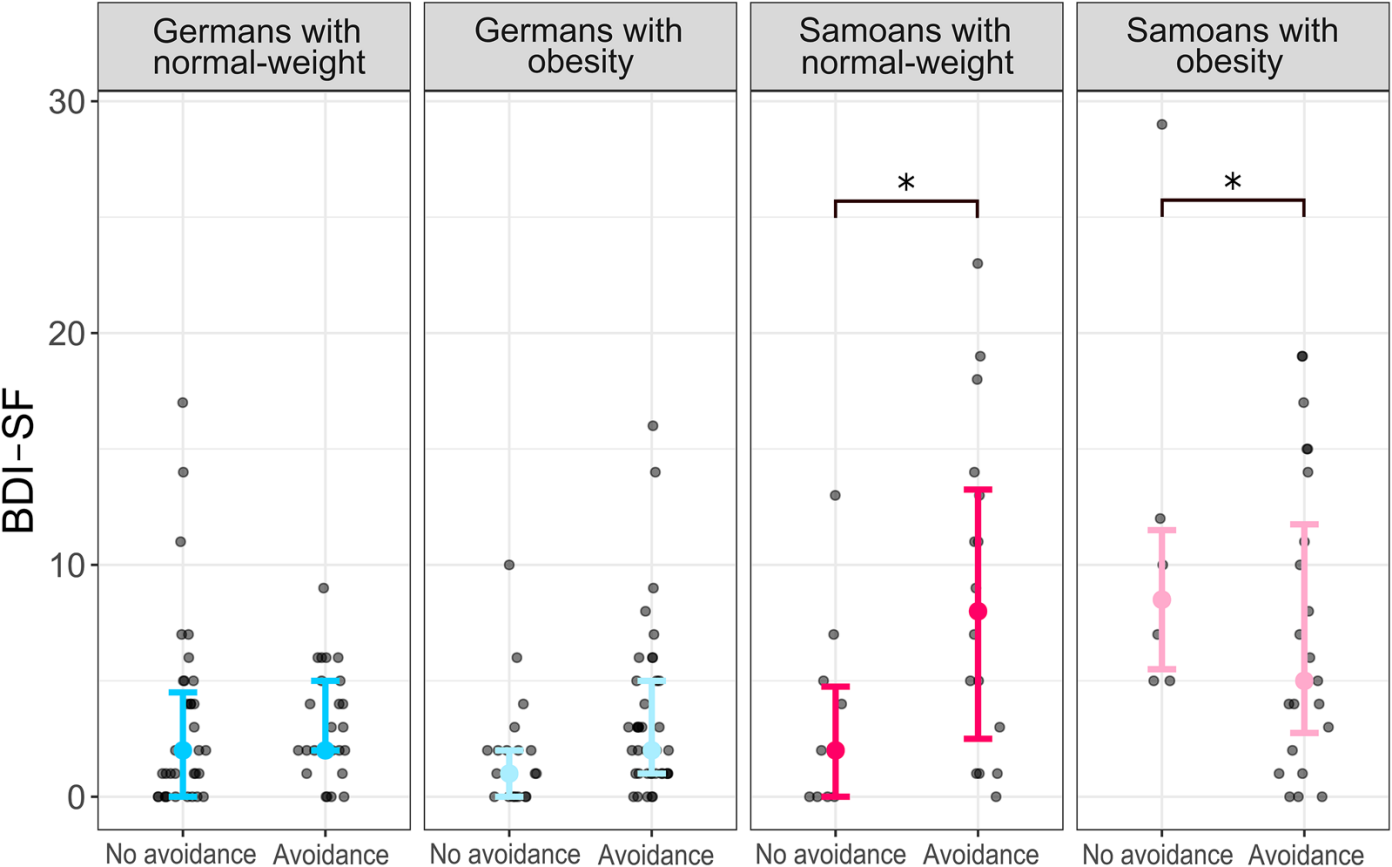
Samoans with normal-weight who did not use avoidance coping reported lower severity of depressive symptoms (*M* = 3.30, *SD* = 4.19) than Samoans with normal-weight who used avoidance coping (*M* = 9.88, *SD* = 7.03). Conversely, Samoans with obesity who did not use avoidance coping reported higher severity of depressive symptoms (*M* = 11.33, *SD* = 9.09) than Samoans with obesity who used avoidance coping (*M* = 6.58, *SD* = 6.03). This effect was absent in the German sample (*F*(1, 166) = 15.427, *p* <.001, η_p_^2^ = 0.085). BDI-SF = Beck’s Depression Inventory short form

For *avoidance* coping, Samoan individuals with normal-weight who were not using avoidance coping reported lower severity of depressive symptoms (*M* = 3.30, *SD* = 4.19) than Samoan individuals with normal-weight who were using avoidance coping (*M* = 9.88, *SD* = 7.03). Conversely, Samoan individuals with obesity who were not using avoidance coping reported higher severity of depressive symptoms (*M* = 11.33, *SD* = 9.09) than Samoan individuals with obesity who were using avoidance coping (*M* = 6.58, *SD* = 6.03). This effect was absent in the German sample (interaction of weight status*location*avoidance coping with BDI-SF: *F*(1, 166) = 15.427, *p* <.001, η_p_^2^ = 0.085; Fig. 3).

### Stress-related eating

**Fig. 4:**
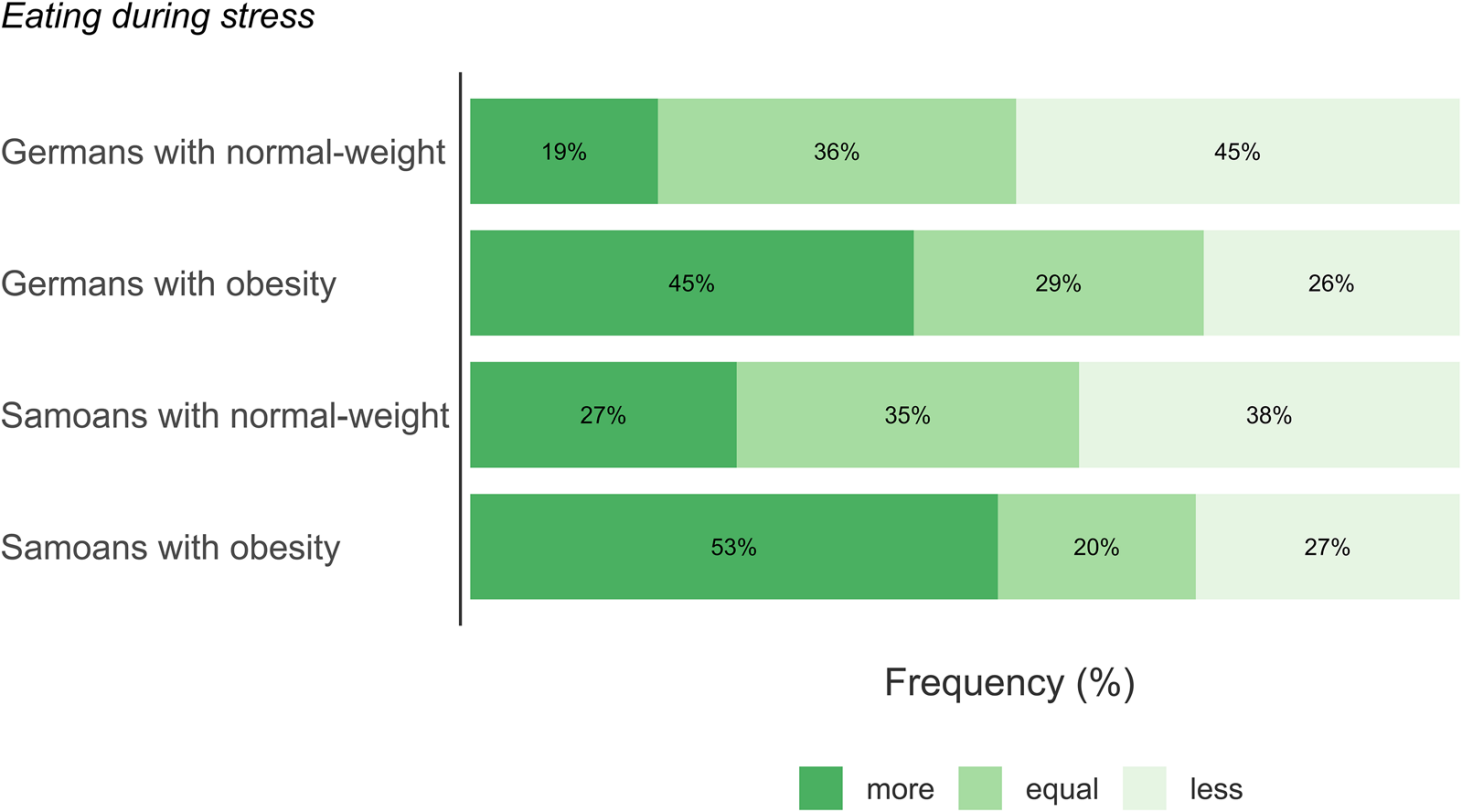
Association between eating during stress and weight status in the German and in the American Samoan sample. In both locations, eating more during stress was associated with weight status (Germany: χ^2^(2) = 9.453, *p* =.009, V = 0.285; American Samoa: χ^2^(2) = 6.497, *p* =.039, V = 0.341)

We found in both cultural samples a significant association between self-reported eating during stress and weight status (Germany: χ^2^(2) = 9.453, *p* =.009, V = 0.285; American Samoa: χ^2^(2) = 6.497, *p* =.039, V = 0.341), indicating that individuals with obesity in both groups tend to eat more during stress, whereas individuals with normal-weight tend to eat less or not change their eating behavior (Fig. 4).

## Discussion

The present study of young adults examined differences in stress coping between a collectivistic (American Samoa) and an individualistic (Germany) group and potential interactions with weight status, perceived stress, or depressive symptoms. In line with our hypothesis, young adult Germans used problem-focused coping more frequently than young adult Samoans, whereas Samoans used avoidance coping more frequently than Germans. In general, depressive symptoms and perceived stress were higher in the Samoan group. We further found that the use of avoidance coping was associated with higher perceived stress among Germans, but with lower perceived stress among Samoans. We also found that Samoans with normal-weight who did not use avoidance coping had lower depressive symptoms than those who did, while Samoans with obesity who did not use avoidance coping had higher depressive symptoms than those who did, but this effect was not observed in the German sample. In both groups, individuals with obesity tended to eat more during stress, whereas individuals with normal-weight tended to eat less or not change their eating behavior. Our results suggest that the two locations differ in their preferred coping strategies during stress, and that the use of these coping strategies might have varying effects on perceived stress and depressive symptoms.

### Comparison of stress coping strategies

In line with the literature, we anticipated a preference for emotion-focused or avoidance coping in the Samoan group and a preference for problem-focused coping in the German group [4, 20, 46]. Our results partially supported our hypothesis: we found more problem-focused coping in the German than the Samoan group and more avoidance coping in the Samoan than the German group. There was no evidence for differences in emotion-focused coping between the two locations and its use was generally high in both locations. Indeed, potential cultural differences in emotion-focused coping are less clear, with some studies reporting more emotion-focused coping in more collectivistic societies [8, 19–22], while others find the opposite for some emotion-focused categories, such as seeking social support [21, 47]. One possible explanation for this variation is that emotion-focused coping encompasses various aspects such as emotion regulation, emotion expression, and/or social support, which single components might vary considerably across cultural groups [21]. A stronger differentiation between different components of coping strategies is therefore recommended in future cross-cultural research. Additionally, some studies might have included strategies classified as avoidance coping in our study in the emotion-focused category (e.g., denial, disengagement, or distraction), partly because stress coping definitions and the distinctions drawn between coping categories vary considerably in the literature [48].

Consistent with our hypothesis, our findings demonstrate that individuals with obesity tend to employ more avoidance coping strategies compared to individuals with normal-weight, regardless of location. Our results are consistent with the literature suggesting that mitigating perceived stress through disengagement, eating, or self-distraction contributes to, or is a factor in maintaining higher weight [1]. As weight loss might reduce the use of avoidance coping in individuals with obesity [49], interventions targeting adaptive stress coping responses might be beneficial for individuals with obesity in both locations. In addition, we showed that individuals with normal-weight were more likely to use emotion-focused coping than individuals with obesity. This aligns with meta-analytic findings of lower emotional awareness and greater suppression of emotional expression in individuals with obesity compared to controls [50].

### Stress coping and perceived stress

Given the adaptive nature of stress coping, our research sought to examine the associations between coping strategies and perceived stress, while recognizing potential variations across the two groups. We also explored potential effects of weight status. In both groups, we found no significant associations between perceived stress and problem-focused or emotion-focused coping. These findings contrast to previous studies [7, 8, 21, 22, 46, 51]. Potential reasons for our findings could be differences in the definitions and scales used for the assessment of these coping strategies between studies. An additional explanation for our findings – which could be accounted for in future studies – may lie in the particular type of stress examined during the interviews (e.g., high work load, examination stress), which might elicit distinct stress coping strategies in these two cultural groups [5].

Further, the use of avoidance coping was associated with lower perceived lack of social recognition and worries in Samoans, but not in Germans. Germans that used avoidance coping, in contrast, exhibited more worries. This finding aligns with a previous study that indicated Polynesian Americans using behavioral disengagement also reported less impairment from stress (Allen & Smith, 2015). Conversely, in individualistic groups, avoidance coping accounted for a higher proportion of variance in stress responses [6]. These results also offer insights into the potential adaptive value of avoidance coping within the Samoan community, particularly concerning social stress. As greater relationship concerns have been found to be a predictor for avoiding seeking social-emotional support in collectivistic societies [47], avoidance coping might be beneficial for maintaining group harmony in the Samoan community.

### Stress coping and depressive symptoms

We hypothesized that the use of different coping strategies would be differentially associated with depressive symptoms in the German and in the Samoan group. We also examined potential interactions with weight status. In both groups, we observed no significant relationships between the use of problem-focused or emotion-focused coping and the severity of depressive symptoms. Although these findings are in contrast with a previous meta-analysis [52], a cross-cultural study also showed no protective effects of problem-focused or emotion-focused coping on depressive symptoms across ethnic groups [53].

Avoidance coping, however, interacted with weight status and location: in the Samoan group, individuals with normal-weight who used avoidance coping had a higher severity of depressive symptoms than those who did not, while the reverse was true for those with obesity. In the German group, avoidance coping was not linked to depression, contrasting prior findings [49]. To date, research examining how stress coping strategies differ between more collectivistic and more individualistic groups, particularly in relation to obesity and depressive symptoms, is sparse. Emerging evidence suggests that African-Americans and some Latino/Hispanic groups with obesity or other unhealthy habits, despite experiencing high levels of stress, report fewer depressive symptoms than those without such habits. This effect was reversed in White Americans [54–56], indicating that unhealthy behaviors might mitigate stress responses in some socio-cultural groups. It is therefore conceivable that avoidance coping, including eating, in Samoan individuals with obesity might attenuate depressive symptoms. Nevertheless, further research is needed to validate these findings.

Also worth noting is the generally higher level of depressive symptoms in the Samoan compared to the German group. Our results align with prior research that identified elevated depressive symptoms in Native Hawaiian and American Samoan populations [57], exceeding European Americans [58], Asian populations [59], and US populations [60]. In addition, suicide rates were found to be particularly high among Pacific Islanders, especially in Samoans [61]. Specifically, interpersonal conflicts were associated with higher levels of depression among Pacific Islanders [58]. It has been hypothesized that the discrepancy between traditional norms and values and the influences of urbanization and westernization might contribute to heightened mental distress [62, 63]. Moreover, the discouragement of openly displaying strong emotions, such as anger, in the Samoan community [64–67] and the emphasis on community needs and social obligations [62, 68], might impede talking about stress or seeking mental health treatment [62]. Those who do seek help might face additional barriers, as mental health services in American Samoa and other Pacific communities have been described as insufficient [62].

### Stress-related eating

Our expectations regarding obesity-related differences in eating during stress were met in both locations. As hypothesized, individuals with obesity demonstrated a tendency to reporting increase food intake during periods of stress, while individuals with normal-weight tended to eat less or maintained their regular eating behavior. This effect has been well studied in Western cultures and supports our observations [69–71]. Our results add to the literature that increased eating under stress, especially in individuals with obesity, might be a robust effect across locations and might be driven less by cultural differences than by biological processes and individual cortisol responses [30, 72].

### Limitations

The first limitation that needs to be considered is the classification of food consumption as avoidance coping, particularly when contextualized within the Samoan culture. In Samoa, consuming and exchanging food are strongly associated with social bonds and relationships and its provision contribute to a sense of social security through supportive social relationships and kinship ties in Samoa [73]. Consequently, the abundance of food and the ability to partake in it can take on a symbolic role, signifying the richness of one’s social support systems. This perspective suggests that eating – by reinforcing a sense of social connection and belonging – may also be an emotion-focused and not solely an avoidance coping strategy in Samoa. However, we intentionally refrained from classifying eating as emotion-focused coping because several studies indicate that emotional eating is associated with deficits in emotion regulation rather than with active regulation of negative affect [74, 75]. Emotional eating has also been linked to greater use of avoidance coping [76], and avoidance coping has been shown to mediate the relationship between negative emotions and emotional eating [77]. Eating as a response to stress often involves distracting from the distress itself and seeking short-term relief without addressing root causes [78]. In our study, we explicitly defined emotion-focused coping as actively engaging with and processing emotions to achieve emotional well-being. Because emotional eating does not meet this criterion and aligns conceptually with avoidance or maladaptive regulation, we classified it accordingly. To ensure methodological consistency between samples, we applied the same classification to the Samoan participants. Nonetheless, as no studies on stress coping strategies in Samoa are available to our knowledge, we cannot exclude the possibility that eating as a coping strategy in this cultural context may not be entirely maladaptive. Future research should revisit this classification given the social meanings attached to food.

In addition, while interviews provide a more comprehensive and in-depth understanding of individuals’ coping concepts and behaviors, they also rely on the individuals’ ability to recall coping strategies. Some participants might underreport their coping behaviors, potentially impacting the accuracy of the data. The advantage of focusing on the most salient coping behavior might result in a lower number of statements and, subsequently, a decrease in diversity. To address these considerations, future studies could adopt a mixed-method approach by combining in-depth interviews with standardized stress coping questionnaires (such as the Brief COPE or Ways of Coping Questionnaire). This would allow researchers to ensure a more comprehensive representation of coping diversity and to evaluate the cultural validity of standardized coping measures.

Further, stress coping definitions, concepts, and measurements, as well as the distinctions drawn between coping categories, vary considerately in the literature, making comparisons challenging [48]. In addition, the comparability of the stress or depression concepts between these two populations remains uncertain. A differential sensitivity to social life events between collectivistic and individualistic societies has been found in other studies [79]. Furthermore, scales validated in Western societies to assess stress and depression (in our study: BDI-SF, PSQ-20, TICS) might inadequately represent culturally relevant aspects among Pacific Islanders such as cultural identity and interpersonal conflicts [58, 80]. These factors might play a crucial role in shaping stress perception and coping responses in the two populations studied. To our knowledge, the cultural validity of these instruments for Polynesian populations has not yet been tested, wherefore we recommend developing and validating culturally specific instruments in future studies.

A significant age difference between the German and Samoan participants was statistically controlled for in all relevant analyses. Although covariate adjustment is an established method in experimental research to account for baseline group differences, it cannot fully eliminate residual confounding. We also recommend including other potential confounders, such as socioeconomic status and acculturation, in future analyses. Further, our sample size was small and imbalanced. While GLM/ANCOVA methods can mitigate bias from imbalanced samples, the smaller Samoan cohort reduces the power to detect small effects and limits subgroup analyses. Replication in larger Pacific Islander samples is therefore essential.

Finally, it is crucial to acknowledge that the presented study’s cross-sectional nature restricts our ability to establish definitive causal relationships and to control for additional potential confounding factors. Longitudinal studies are needed to determine the temporal order of the associations found in this study.

### Implications

The results of this study are consistent with previous discussions that emphasized the important role of the socio-cultural environment in shaping stress coping strategies and their adaptive effects on depressive symptoms and perceived stress. However, these aspects remain insufficiently represented in current stress coping research. Consequently, future studies should therefore integrate the socio-cultural background and adaptive coping responses of participants as essential factors in their analyses.

Our findings can, for instance, be of use for the development of culturally appropriate interventions that align with the distinct coping profiles observed in our study. It is essential to recognize that stress management approaches cannot be universally applied across all backgrounds and should instead be thoughtfully tailored to the unique coping profiles of each population. Given that avoidance coping was associated with a higher level of perceived stress in our German sample, interventions could focus on helping individuals recognize and reduce maladaptive avoidance. For individuals from Samoa, an intervention that bluntly discourages avoidance coping could be culturally inappropriate and potentially harmful, as our results suggest an adaptive function. A more effective approach might focus on addressing the higher overall levels of depressive symptoms we found in this group, perhaps by introducing culturally-grounded strategies that allow for personal distress to be managed without disrupting community relationships. Taking these differences into account when developing interventions can improve adherence to stress management programs, enhance treatment outcomes, and promote long-term mental and physical well-being.

Independent of location, we found that individuals with obesity tended to more often employ avoidance coping. This observation suggests that this effect might be relatively stable across individuals with obesity in different contexts. Although avoidance coping can reduce the immediate impact of stressful situations and might have short-term protective effects on mental well-being, it may also carry negative consequences for long-term physical health due to unhealthy behavioral responses. We therefore propose that weight loss interventions should specifically address and target avoidant stress coping behaviors.

### Conclusion

Our findings add to the understanding of how the socio-cultural environment shapes the impact of different coping strategies on perceived stress or depressive symptoms. Notably, our study reveals that specific coping strategies cannot be universally deemed superior to others in responding to stress; rather, their effectiveness varies depending on the socio-cultural context. While we found that avoidance coping was more prevalent among individuals with obesity in both locations, the comparison between the Samoan and German group showed distinctive coping patterns during stress that were linked to varying effects on perceived stress and depressive symptoms. In the more collectivistic Samoan culture, the use of avoidance coping appeared to alleviate perceived stress, particularly concerning social stressors. Conversely, in the more individualistic German culture, we observed an opposite effect. These results underscore the importance of considering the socio-cultural background when examining stress coping responses and their implications for mental well-being.

## Data Availability

The data cannot be shared openly to protect study participant privacy. Data is provided on request.
